# PVA-Based Hydrogels Loaded with Diclofenac for Cartilage Replacement

**DOI:** 10.3390/gels8030143

**Published:** 2022-02-24

**Authors:** Ana C. Branco, Andreia S. Oliveira, Inês Monteiro, Pedro Nolasco, Diana C. Silva, Célio G. Figueiredo-Pina, Rogério Colaço, Ana P. Serro

**Affiliations:** 1 CQE—Centro de Química Estrutural, Instituto Superior Técnico, Universidade de Lisboa, Av. Rovisco Pais 1, 1049-001 Lisbon, Portugal; ana.branco@tecnico.ulisboa.pt (A.C.B.); andreia.oliveira@tecnico.ulisboa.pt (A.S.O.); maria.ines.monteiro@tecnico.ulisboa.pt (I.M.); pedro.nolasco@tecnico.ulisboa.pt (P.N.); dcsilva.aquarius@gmail.com (D.C.S.); 2 CiiEM—Centro de Investigação Interdisciplinar Egas Moniz, Instituto Universitário Egas Moniz, Quinta da Granja, Monte de Caparica, 2829-511 Caparica, Portugal; 3 CDP2T—Centro de Desenvolvimento de Produto e Transferência de Tecnologia, Escola Superior de Tecnologia de Setúbal, Instituto Politécnico de Setúbal, Estefanilha, 2910-761 Setúbal, Portugal; celio.pina@estsetubal.ips.pt; 4 IDMEC—Departamento de Engenharia Mecânica, Instituto Superior Técnico, Universidade de Lisboa, Av. Rovisco Pais 1, 1049-001 Lisbon, Portugal; rogerio.colaco@tecnico.ulisboa.pt; 5 CeFEMA—Centro de Física e Engenharia de Materiais Avançados, Instituto Superior Técnico, Universidade de Lisboa, Av. Rovisco Pais 1, 1049-001 Lisbon, Portugal

**Keywords:** cartilage replacement, hydrogel, polyvinyl alcohol (PVA), polyacrylic acid (PAA), polyethylene glycol (PEG), annealing, diclofenac release, mechanical behavior, friction behavior, irritability

## Abstract

Polyvinyl alcohol (PVA) hydrogels have been widely studied for cartilage replacement due to their biocompatibility, chemical stability, and ability to be modified such that they approximate natural tissue behavior. Additionally, they may also be used with advantages as local drug delivery systems. However, their properties are not yet the most adequate for such applications. This work aimed to develop new PVA-based hydrogels for this purpose, displaying improved tribomechanical properties with the ability to control the release of diclofenac (DFN). Four types of PVA-based hydrogels were prepared via freeze-thawing: PVA, PVA/PAA (by polyacrylic acid (PAA) addition), PVA/PAA+PEG (by polyethylene glycol (PEG) immersion), and PVA/PAA+PEG+A (by annealing). Their morphology, water uptake, mechanical and rheological properties, wettability, friction coefficient, and drug release behavior were accessed. The irritability of the best-performing material was investigated. The results showed that the PAA addition increased the swelling and drug release amount. PEG immersion led to a more compact structure and significantly improved the material’s tribomechanical performance. The annealing treatment led to the material with the most suitable properties: besides presenting a low friction coefficient, it further enhanced the mechanical properties and ensured a controlled DFN release for at least 3 days. Moreover, it did not reveal irritability potential for biological tissues.

## 1. Introduction

Osteoarthritis (OA) is the most common chronic joint condition, affecting more than 300 million people worldwide [1]. Its prevalence is expected to increase significantly in the coming years due to the aging of the population and issues related with obesity, two characteristics of Western societies [2]. It causes pain and severe limitations in joint function, leading to disability and a decrease in patients’ quality of life. Several factors condition the type of treatment applied, e.g., the patients’ age and gender, the location of the cartilage defect, its extent and depth [3]. For less severe cases, management of OA involves physiotherapy and/or the prescription of drugs to prevent or minimize disease progression [4], whereas severe clinical cases may require regenerative surgical procedures or partial/total replacement of the affected tissues [5].

Various polymeric biomaterials have been proposed to act as cellular scaffolds and support the growth of newly formed tissue [6,7,8] or to substitute areas of damaged cartilage [9]. Hydrogels are 3D hydrophilic polymeric matrices that are commonly used in these applications because of their interesting features, e.g., chondrogenic potential, ready transduction of mechanical loads, the possibility of in situ scaffold formation, and high swelling capacity [6,10,11]. Polyvinyl alcohol (PVA) hydrogels have been extensively studied as cartilage replacement materials due to their biocompatibility, chemical stability, adhesive capability, simple preparation, and ability to be tailored [12,13]. PVA hydrogels are typically obtained by dissolving the polymer in water and physically crosslinking through the establishment of hydrogen bonds between the OH groups of its molecular chains [12]. Chemical crosslinking methods may also be used. However, chemical crosslinkers are often toxic and may affect the material’s biocompatibility [14]. These hydrogels can be prepared using injection molding, irradiation, cast drying, and freeze–thawing (FT) [12,15]. FT produces highly porous, spongy, and rubbery materials through a cyclic process of freezing and thawing. This method promotes chain reticulation via liquid–liquid phase separation, hydrogen bonding, and crystallite formation [16,17]. FT PVA hydrogels generally produces a greater swelling capacity but a lower elastic modulus and less resistance to wear than natural cartilage. Although their properties may be improved by changing the number and duration of FT cycles [12], alternative reinforcement strategies have been attempted to enhance the materials’ mechanical performance. These include the incorporation of other polymers of synthetic (e.g., poly(lactic-co-glycolic acid, poly(ethylene glycol) diacrylate, polyvinylpyrrolidone) or natural origin (e.g., collagen, hyaluronic acid, chitosan, alginate) [18,19]; nano/microparticles (e.g., silica and hydroxyapatite) [20,21] and other differentiated structures (e.g., graphene sheets) [22]; and the use of physical treatments, such as thermal annealing [23].

The high swelling capacity of hydrogels makes them ideal systems to encapsulate bioactive molecules or hydrophilic drugs that can be locally released into synovial joints, reducing the risks of drug degradation commonly associated with oral and intravenous administration, side effects, and required doses [11,24]. Several authors have been working on the development of controlled release systems capable of improving cartilage tissue regeneration [25,26] or relieving inflammatory symptoms after orthopedic surgeries [27,28,29,30]. Non-steroidal anti-inflammatory drugs (NSAIDs), such as diclofenac (DFN), are usually prescribed for oral administration in the first few days after these interventions. In addition to their anti-inflammatory action, they also present analgesic and antipyretic properties [31], acting as inhibitors of the activity of cyclooxygenase enzymes (COX-1 or COX-2), which are involved in the synthesis of key biological mediators of inflammation [32,33]. An ideal drug release system must have predictable release kinetics and maintain an effective drug concentration in the tissues over an appropriate period. Drug release depends on the material’s composition and porosity, which control, respectively, the chemical and steric interactions between the drug molecules and the polymer network [11]. Although several strategies can be applied to load the drug into the hydrogels and control its release (incorporation of drug-loaded nanoparticles or other colloidal nanostructured systems, molecular imprinting, addition of ligands/functional monomers to the polymeric matrix, or incorporation of molecules that work as diffusion barriers [34]), soaking them into the drug solution is the most straightforward and less expensive loading method, but usually results in an initial burst and a limited release period [35]. However, depending on the drug and polymeric matrix, suitable drug release profiles may be achieved.

The main objective of this work was to develop a cartilage replacement material based on PVA and produced using FT with an adequate mechanical and tribological performance and the ability to control the release of DFN in the immediate postoperative period. Poly(acrylic) acid (PAA) was added to PVA to enhance the drug release profile [36]. PAA is a highly soluble biodegradable polymer that has been widely used in the biomaterials field as a drug carrier. In fact, due to its hydrophilicity and high propensity to absorb water, it aids the drug’s entrance into the hydrogels. Additionally, it presents good bioadhesive properties, increasing the drugs’ residence time in the tissues and their penetration and, consequently, their bioavailability [37]. The PVA/PAA hydrogels were then immersed in poly(ethylene glycol) (PEG), which is a biocompatible, nonionic, hydrophilic, flexible, nontoxic, and nonimmunogenic polyether that is widely used for lubrication purposes and has exceptional resistance to protein adsorption [37,38,39,40]. It can affect not only the solubility, mechanical, and thermal properties of the polymeric systems containing it but also their crystallinity and viscosity [40]. Choi et al. [41] found that PEG immersion of PVA/PAA hydrogels prevented their embrittlement and resulted in a higher surface lubricity. Finally, the PVA/PAA+PEG hydrogel was submitted to an annealing treatment, which was expected to increase the material’s crystallinity by promoting additional crosslinking, leading to improved mechanical and tribological properties [23,30]. The effect of each of these steps on the hydrogels’ morphology, swelling, water content, wettability, compressive mechanical properties, and friction coefficient was evaluated. The swelling, water content, wettability, rheological, and compressive behavior of the different hydrogels was also compared with that of porcine cartilage or with literature values for natural cartilage. The release profiles of DFN were studied under sink conditions. Lastly, an irritability test was performed on the most promising material.

## 2. Results

### 2.1. Morphology

**Figure 1:**
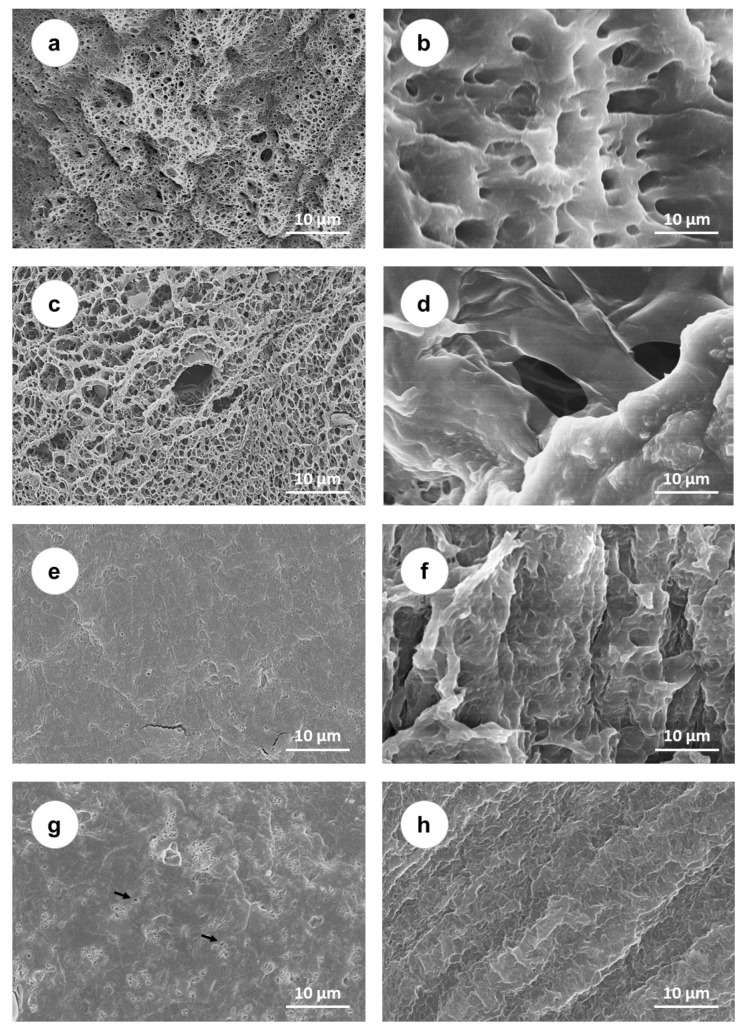
SEM micrographs of PVA, PVA/PAA, PVA/PAA+PEG, and PVA/PAA+PEG + A samples: surface morphology (**a**,**c**,**e**,**g**, respectively) and cross-cut section (**b**,**d**,**f**,**h**, respectively) (scale bar: 10 µm). Arrows show examples of pore-like clusters.

SEM images of the samples’ surfaces and cross-cut sections are shown in Figure 1. Both the PVA (Figure 1a,b) and PVA/PAA (Figure 1c,d) hydrogels presented a porous structure, but the addition of PAA seemed to lead to the formation of larger pores. In both cases (PVA and PVA/PAA), a much less lacy structure was observed in the materials’ bulk. The breaking of the samples may have contributed to this fact. Samples with PEG incorporated (Figure 1e,f) exhibited smoother and more uniform surfaces and the bulk structure was essentially non-porous. After annealing (Figure 1g,h), some changes were observed on the surface, namely, the appearance of small pore-like clusters, but the bulk remained non-porous.

### 2.2. Water Uptake

**Figure 2:**
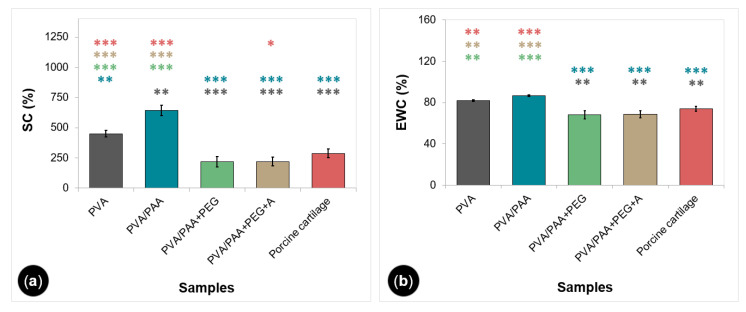
Swelling capacity (**a**) and equilibrium water content (**b**) of the hydrogels and porcine cartilage. Error bars are the ± standard deviations, *n* = 3. Data were analyzed using ANOVA (*p* < 0.0001) and Tukey’s HSD test. Statistical differences are indicated by asterisks (* *p* < 0.05, ** *p* < 0.005, *** *p* < 0.0005).

The SC and EWC of gels and porcine cartilage are shown in Figure 2. PVA and PVA/PAA had similar values of absorbed water (82% ± 1% and 87% ± 1%), but their swelling ratio was statistically different (451% ± 26% and 643% ± 42%). PEG-doped and annealed samples presented lower but comparable values of swelling (218% ± 41% and 221% ± 35%) and water content (68% ± 4% and 69% ± 4%), with a reduction of about 50% and 16%, respectively, compared to the PVA values. Natural cartilage led to values similar to those obtained for PVA/PAA+PEG and PVA/PAA+PEG+A.

### 2.3. Wettability

**Figure 3:**
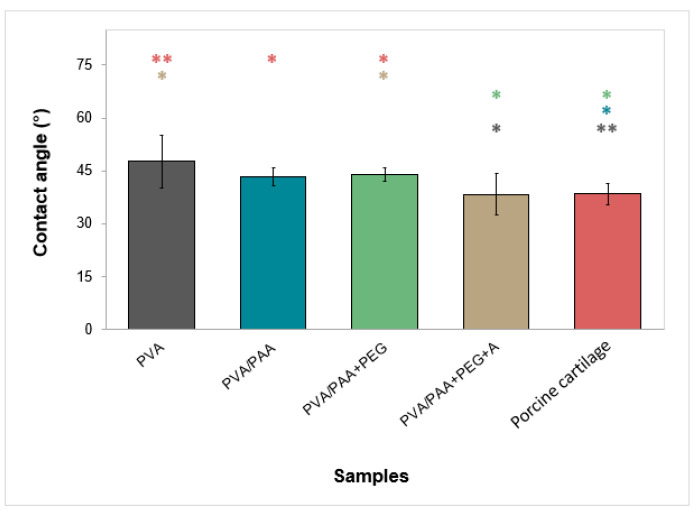
Water contact angles with hydrogels. Error bars are the ± standard deviations, *n* = 8. Data were tested using Welch’s ANOVA (*p* > 0.05). Statistical differences are indicated by asterisks (* *p* < 0.05, ** *p* < 0.005).

The results of the wettability measurements of the materials’ surfaces are given in Figure 3. All hydrogels were hydrophilic, with water contact angles between 38° and 48°. Annealed samples presented the lowest contact angle, similar to that obtained for the porcine cartilage.

### 2.4. Mechanical Behavior

**Figure 4:**
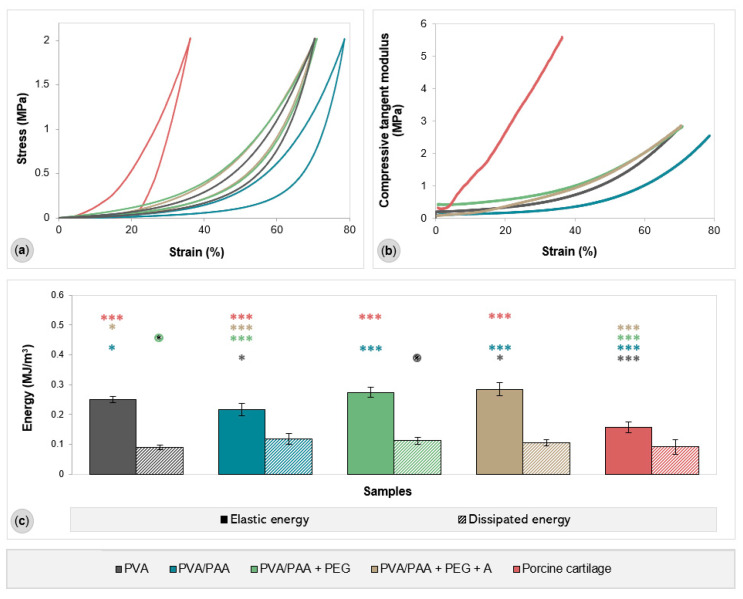
Typical compression stress–strain curves (**a**), compressive tangent modulus (**b**), and elastic and dissipated energies (**c**) of the samples. Error bars are the ± standard deviations, *n* = 5. The elastic energy data were analyzed using ANOVA (*p* < 0.0001) and Tukey’s HSD test. The remaining data were evaluated using Welch’s ANOVA (*p* < 0.05) and Dunnett’s T3 test. Statistical differences are indicated by asterisks (* *p* < 0.05, *** *p* < 0.0005).

Representative compressive stress–strain curves of all tested materials are provided in Figure 4, together with their compressive tangent modulus for the entire strain range, elastic energy (area of the hysteresis loop), and dissipated energy (total area under the unloading curve). All hydrogels showed a non-linear stress–strain relationship, meaning that all samples, including the porcine cartilage samples, exhibit viscoelastic behavior. The addition of PAA to PVA led to a decrease in the compressive modulus for the entire strain range, with this hydrogel being the most compressible of all (it reached a strain of about 80%). In contrast, porcine cartilage proved to be stiffer and less compressible, with a higher modulus of elasticity and a maximum compressive strain of 36%. PEG-doped and annealed hydrogels presented a compressive behavior similar to PVA. As for elastic energy, porcine cartilage showed the lowest value (0.14 MJ/m³), while the lowest among the hydrogels was found for PVA/PAA (0.22 MJ/m³). No statistically significant differences were observed for the dissipated energy, except between PVA and PVA/PAA+PEG.

### 2.5. Rheological Behavior

**Figure 5:**
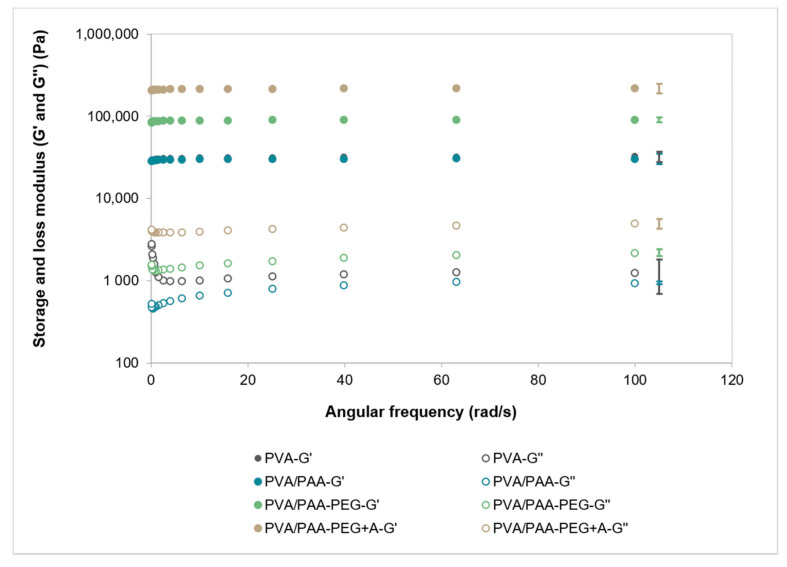
Variation of the storage modulus (G’) and loss modulus (G’’) during an angular frequency sweep for all studied hydrogels. Error bars are the ± standard deviations, *n* = 4.

The angular frequency curves obtained for the different hydrogels are shown in Figure 5. In all cases, G’ was more than one order of magnitude higher than G’’ throughout the whole frequency range. PVA and PVA/PAA showed the lowest G’ and G’’ values. The addition of PEG resulted in an increase of the elastic contribution (G’), which was even more significant after annealing. A similar tendency was found for G’’, but at a much lower scale.

### 2.6. Friction Coefficient

**Figure 6:**
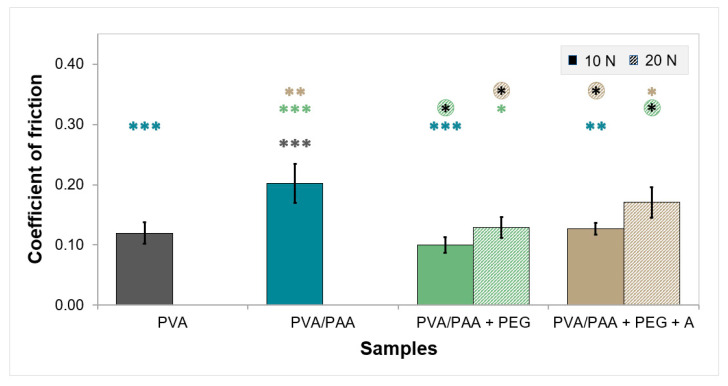
Friction coefficients of the samples tested against SS 316L in PBS solution under a load of 10 or 20 N. Error bars are the ± standard deviations, *n* = 3. Pairwise comparisons involving at least one group assayed at 20 N were done using the independent samples *t*-test. All other comparations were performed using ANOVA (*p* < 0.0005) and Tukey’s HSD test and refer to comparisons with results obtained with load 10 N and 20 N, respectively. Statistical differences are indicated by asterisks (* *p* < 0.05, ** *p* < 0.005, *** *p* < 0.0005).

Figure 6 provides the average CoF values for samples tested in PBS solution under 10 and 20 N of loading. PVA and PVA/PAA gels did not withstand 20 N of load and, therefore, no values of CoF could be obtained. The friction behavior of PEG-doped and annealed samples under the 10 N load was identical to that of the PVA gels. However, CoF rose about 1.7-fold for PVA/PAA. For the 20 N load, the CoF values increased slightly.

### 2.7. Drug Loading and Release

The total amount of DFN loaded by the samples is depicted in Table 1.

PVA loaded the lowest amount of drug, while PVA/PAA loaded the highest. The immersion in PEG led to an intermediate value. Annealing barely affected the loading capacity (*p* = 0.676).

**Figure 7:**
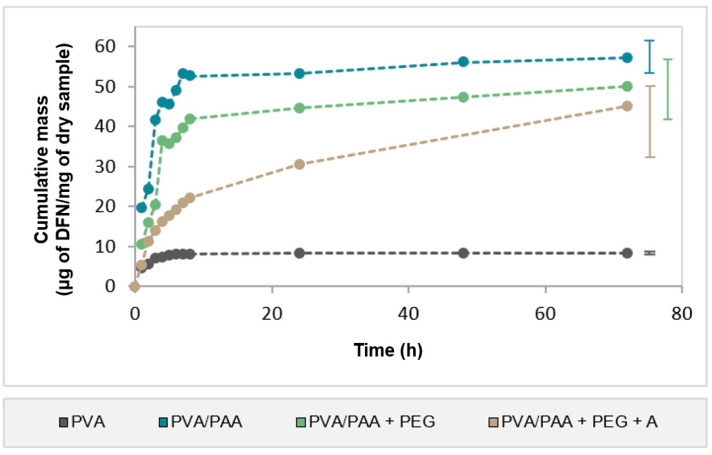
Cumulative drug release profiles from samples loaded with DFN. Error bars are the ± standard deviations, *n* = 4.

Cumulative DFN release profiles from all samples are provided in Figure 7. The drug release from PVA samples was almost completed in 6 h. The addition of PAA to PVA led to an increase in the total amount of drug released by about 5.8-fold. Its release profile presented an initial burst and, similarly to PVA, the release occurred mainly in the first few hours. Immersion in PEG reduced the total amount of DFN released but did not change the profile shape. Finally, annealing further reduced the amount of drug released but led to a controlled profile, ensuring a prolonged release during at least 72 h. The drug released amount by this sample was about 55% of the drug loaded (Table 1).

When comparing the amount released for all samples, it was found that a significant amount of drug remained in the hydrogels’ matrix, particularly for PVA/PAA (54.3%).

### 2.8. HET-CAM Test

**Figure 8:**
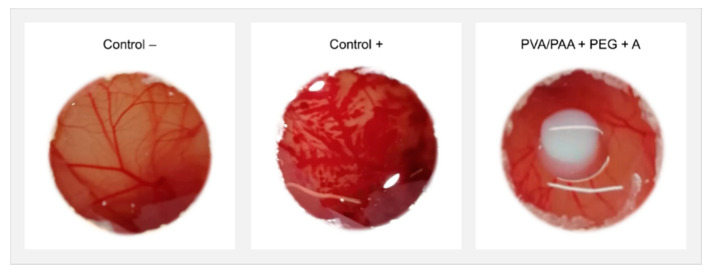
CAM images after incubating the annealed samples loaded with DFN for 5 min. Reaction with negative and positive controls are also shown.

The observation of CAM of hen’s eggs after 5 min of exposure to annealed samples loaded with DFN did not show signs of hemorrhage, lysis, or coagulation occurring during the test, similar to what happened with the negative control (Figure 8). In contrast, the positive control led to a severely irritating reaction, with bleeding, disintegration of blood vessels, and protein denaturation.

## 3. Discussion

In this work, a set of PVA-based hydrogels were studied with the purpose of developing materials with adequate properties to be used as cartilage substitutes and simultaneously release an anti-inflammatory drug, namely, DFN. PVA and a blend of PVA/PAA were prepared. The latter was subsequently immersed in PEG (PVA/PAA+PEG) and thereafter submitted to an annealing treatment (PVA/PAA+PEG+A).

The morphological analysis of the materials (Figure 1) showed that, as expected, the PVA hydrogel prepared using FT presented a porous structure. As referred in other works [17,42], this method led to PVA chains physically crosslinking through a mechanism that involves liquid–liquid phase separation, the establishment of inter- and intramolecular hydrogen bonds, and the formation of crystallites that act as knots between the polymer chains segments. Freezing leads to the growth of ice crystals, which move the polymer chains away, originating zones with different polymer contents. In the polymer-rich areas, crystallites growth occurs during thawing, water forms hydrogen bonds with the OH group of PVA and acts as a swelling agent inducing the appearance of pores [43]. The addition of PAA leads to an increase in porosity. According to Sanchez et al. [44], PVA and PAA chains are aligned and, upon swelling, partial leaching of PAA occurs, leading to an increase in porosity. The immersion in PEG and its diffusion into the hydrogel is known to cause the removal of water [45,46]. Furthermore, it can form extra hydrogen bonds with the hydroxyl groups from PVA [47]. After 5 days at 60 °C, it was observed that the hydrogels’ porous structure was lost, which might have been due to the chains’ mobility resultant from this drying process and to the establishment of new bonds. Posterior annealing treatment at 120 °C should increase the mobility of the chains, allowing their alignment and folding to form new micro-crystallites [15]. SEM analysis revealed that the hydrogel remained essentially non-porous, with the appearance of some irregular features on the surface (pore-like clusters).

The study of the swelling capacity of the hydrogels (Figure 2a) showed that the addition of PAA to PVA led to an increase in the water content in the hydrated hydrogel. This could be attributed to the presence of carboxyl groups of PAA, which can establish hydrogen bonds with water molecules, aiding their absorption into the hydrogel matrix. The addition of PEG led to a deswelling, as referred to above. This, together with the substantial decrease in porosity, might explain the significant reduction in swelling capacity. The annealing treatment did not significantly affect the swelling ability. Results reported in the literature [48] show that, in general, swelling decreases with the increase in the annealing time due to a higher crosslinking density formed in the hydrogels, which restricts the extensibility of the polymer chains caused by swelling. However, some authors did not find substantial changes in the water absorption capacity above certain annealing times. For example, Park et al. [48] observed that annealing PVA/PEG hydrogels at 120 °C for more than 30 min barely alters the degree of swelling. Therefore, it can be inferred that the previous drying at 60 °C over five days after immersion in PEG should have had a major role in the structural changes that determine the hydrogel’s swelling behavior. The swelling capacity of porcine cartilage was determined for comparison purposes. The obtained average value was not statistically different from the ones obtained for PVA/PAA+PEG and PVA/PAA+PEG+A. Since the water content of the natural joint cartilage is usually referred to in the literature in terms of EWC, this parameter was also estimated for the studied hydrogels (Figure 2b). The obtained values fell within the range of those reported by other authors (65–80%) [49].

Concerning wettability, the studied materials did not present significant differences between them. As expected, all materials showed hydrophilic behavior (water contact angles between 42 and 47°) since the polymers used in their preparation are hydrophilic [50,51,52]. Wettability depends on several factors, such as roughness, porosity, surface heterogeneity, chemical composition, and moisture content. Understanding the effect of these different factors is a complex task since they can act in opposite directions. The obtained values were lower than those reported in the literature (76–103° [53]). Comparison of the obtained results with such values is not straightforward since they are frequently obtained using the sessile drop method in which the surface is not in equilibrium with the aqueous medium, but in this work, the captive bubble method was used to better mimic the in vivo conditions since the samples were immersed in aqueous media. Moreover, the obtained values were the same order of magnitude as those measured in this work for porcine cartilage (38 ± 3°). The lower values found for natural cartilage through this method resulted from conformational changes in the phospholipidic layer of the cartilage surface: in the presence of water, the lipid bilayer has a hydrophilic character, while in a dry environment, it changes to a monolayer with hydrophobic behavior [53].

The mechanical properties of the studied hydrogels were accessed using compressive tests. The stress–strain curves show the typical behavior observed for the studied materials. Hydrogels are biphasic materials consisting of a fluid phase and a solid matrix. At the beginning of the compressive test, when the load is applied, the resistance to deformation is mainly due to the fluid pressurization. As the fluid exits the hydrogel due to compression, the load is transferred to the solid phase, and the slope of the curve increases significantly. PVA/PAA exhibited the lowest compressive modulus, which was associated with the higher amount of water present in the sample and the high porosity of the hydrogel, which allowed for the easy release of water. In addition, PAA reduced the PVA chains’ intermolecular interactions decreasing the material’s stiffness. PVA/PAA+PEG and PVA/PAA+PEG+A gave rise to hydrogels with the highest compressive modulus, most likely due to their lower porosity and water content, as well as stronger interactions between the molecular chains and/or formation of microcrystallites.

The rheological measurements showed that all the hydrogels displayed viscoelastic behavior, showing viscous and elastic characteristics simultaneously. The materials presented predominantly elastic behavior since the storage modulus was dominant over the loss modulus (G’ > G’’) for all analyzed frequencies. This means that the hydrogels are stable during storage [54]. As both moduli were practically independent of the studied frequency range, this also reveals a stable gel-like behavior of the materials. Although values of the same order of magnitude have been found for G’ and G’’ by other authors for PVA hydrogel [55], they are quite different depending on the hydrogel composition, the preparation protocols, and measurement methodology [56]. It must be stressed that the values found in the literature for natural cartilage are about one order of magnitude superior for G’ and two orders for G’’ [57,58]. The addition of PAA did not lead to significant changes in both moduli. However, after immersion in PEG, a noticeable increase was observed, especially for G’, indicating that the energy storage capability of the hydrogel became higher. This was even more pronounced after annealing, which was in agreement with the improvement found in the mechanical properties.

The study of the friction coefficient (CoF) of cartilage replacement materials is crucial, as it evaluates the resistance to movement of the joint interface. The friction of hydrogels depends on many factors, such as the materials’ ability to suffer deformation, EWC, hydrophilicity, roughness, and adhesion to opposing surfaces. The results showed that the lower the compressive modulus, the higher the CoF due to the higher deformability of the material. This increased the contact area, which enhanced the adhesive component of the tangential force and resulted in a higher counter body penetration depth into the hydrogel that impaired the sliding, explaining the higher value of CoF observed for PVA/PAA. The higher water amount in this hydrogel should have contributed to a decrease in the CoF relative to PVA. However, its higher deformation capacity and porosity played a more relevant role, increasing the friction. The remaining hydrogels presented CoF values that were similar to each other. As expected, increasing the applied load resulted in higher CoF values, which was also due to the increase in penetration depth and contact area. It should be stressed that all CoF values were in the range of those found in the literature for articular cartilage tested against stainless steel [59,60].

To evaluate the potential of the developed materials to be used as drug delivery platforms, loading and release studies were performed with DFN. Significant differences in the loading capacity of the samples were observed. PVA loaded the lowest amount of DFN (14.6 ± 0.9 μg DFN/mg dry sample) and released about 58% of that drug in less than 6 h. This hydrogel has a porous structure with free spaces that can contribute to the fast loading and release of the drug. Other authors [61] reached similar conclusions regarding the effect of porosity on the drug release from hydrogels. The addition of PAA to PVA led to a notable increase in the amount of drug being loaded and released, which should be related to the presence of hydrophilic carboxylic side groups (–COOH), which was responsible for the extensive swelling and greater porosity. A similar tendency was observed by Sanli et al. [62] in PVA membranes after PAA incorporation. In a study with hydrogels containing acrylic acid, Jalil et al. [63] performed XRD analysis and found no apparent interaction between DFN and the hydrogels. This may explain the fast release of the drug, which was almost completed in 8 h, similarly to PVA. Concerning PVA/PAA+PEG, a significant decrease in the amount of drug loaded was observed relative to PVA/PAA (lower by 37.9%, Table 1). This may have been due to structural changes induced by PEG in the material, which led to a decrease in its porosity and swelling capacity. However, the total amount of DFN released after 72 h was only 15.6% lower than that found for PVA/PAA, although it remained significantly above that obtained with the PVA hydrogel. PVA/PAA+PEG showed drug release kinetics similar to PVA/PAA, which may be related with the lack of chemical interactions between the drug and PEG, as demonstrated by Chen et al. [64]. The annealing treatment barely affected the DFN loading capacity of the hydrogel. However, it induced significant changes in the drug release behavior: a sustained release of DFN was observed, which lasted for at least 72 h. This may be attributed to the hydrogel’s non-porous structure, together with the higher degree of crosslinking that slowed down the DFN release. This also may explain the lowest percentage of DFN released compared to the non-annealed hydrogel (55.3% vs. 64.3%, Table 1).

In order to infer the drug release mechanism for the hydrogel that led to the best release behavior (PVA/PAA+PEG+A), the Korsmeyer–Peppas model [65] was fitted for the first 60% of its fractional release, as follows:

(1) $$\frac{M_{t}}{M_{\infty }}=Kt^{n}$$

where *M_t_* and *M_∞_* are the masses released at time t and at infinite time (obtained via extrapolation using TableCurve software), respectively; *n* is the diffusional exponent; and *K* is the pseudo-kinetic constant. The fitting led to *n* = 0.4 and *K* = 0.03, with a correlation coefficient of R^2^ = 0.96, demonstrating that the model adequately described the drug release behavior. When *n* < 0.5, a pseudo-Fickian behavior with a diffusion-controlled mechanism occurs [66], leading to short release times [11]. However, changes in the mesh size of the hydrogel may affect the drug diffusion inside the polymeric network. The hydrogel’s mesh size can be estimated based on the classical theory of rubber elasticity using the following equation [11,67,68]:

(2) $$r_{mesh}={\left(\frac{6RT}{\pi N_{AV}G}\right)}^{1/3}$$

where *R* is the gas constant, *T* is the absolute temperature, *N_A_* is Avogadro’s number, and *G* the shear modulus, which can be calculated using

(3) $$G=\sqrt{{G^{\prime }}^{2}+{G^{\prime \prime }}^{2}}$$

Taking into consideration the fact that G’’ was much lower than G’ (see Figure 5), *G* can be approximated as G’. From the values obtained for PVA/PAA+PEG and PVA/PAA+PEG+A, the correspondent mesh sizes are 4.4 nm and 3.3 nm, respectively.

The hydrodynamic radius of DFN in PBS was determined by Pimenta et al. [69] from the measurement of the bulk aqueous diffusion coefficient using pulsed gradient spin-echo and Stokes–Einstein theory and was found to be 0.25 nm. The lower value of the ratio mesh size/drug size for PVA/PAA+PEG+A compared to PVA/PAA+PEG may explain the slower DFN release rate.

The global analysis of the results led to the choice of PVA/PAA+PEG+A as the most promising material for cartilage replacement since it presented the best mechanical properties and friction behavior, as well as the best drug release profile. Therefore, this material was subjected to biological tests to predict the potential for irritability that it could induce in biological tissues. The absence of hemorrhage, lysis, or coagulation in the HET-CAM tests resulted in an irritation score of 0. This result was expected since, in previous studies, it was demonstrated that PVA-based hydrogels loaded with diclofenac did not induce cytotoxic effects [30].

## 4. Conclusions

In this work, PVA-based hydrogels were produced to be used as cartilage replacement materials. Aiming to improve their tribomechanical behavior and to provide them with the ability to act as drug release platforms of the anti-inflammatory drug DFN, several approaches were followed:

(1) PAA was added to the formulation of hydrogels to obtain PVA/PAA samples;
(2) PVA/PAA was further immersed in PEG to obtain PVA/PAA+PEG samples;
(3) PVA/PAA+PEG was annealed at 120 °C, giving rise to PVA/PAA+PEG+A samples.

The addition of PAA resulted in a porous material with slightly larger pores than PVA, greater swelling capacity, lower mechanical properties, and a higher CoF. Furthermore, it led to a significant increase in the amount of drug released, but as with PVA, the release was completed in a few hours. The lower mechanical resistance and the higher water absorption capacity played a determinant role in the frictional and drug release behavior, respectively.

The immersion of PVA/PAA in PEG led to dehydration of the samples and established interactions with the PVA chains, which were responsible for the compaction of the structure and improvements in the mechanical properties. In turn, these last two factors explained the decrease in CoF relative to PVA/PAA. Concerning the DFN release, the lower porosity and swelling capacity contributed to the decrease in the amount of drug released compared to the hydrogel before PEG immersion. However, release the kinetics remained similar.

After the annealing treatment, the hydrogels kept a non-porous structure, with similar swelling capacity and CoF compared to PVA/PAA+PEG. However, the higher crosslinking due to the increased degree of crystallinity led to a significant improvement in the mechanical properties and a more sustained release of DFN, which lasted for at least 3 days. The material proved to be a non-irritant to biological tissues.

Overall, the final hydrogel (PVA/PAA+PEG+A), obtained through a set of sequential steps, revealed a superior tribomechanical behavior that approximated that of natural cartilage. Moreover, the material showed a high potential to ensure a sustained release of the anti-inflammatory DFN at the local level in the post-surgical period without inducing harmful effects to biological tissues. Further studies on the wear/fatigue behavior of this material, as well as on its ability to prevent an inflammatory response, would be needed to prove its efficacy for cartilage substitution.

## 5. Materials and Methods

### 5.1. Materials

Polyvinyl alcohol (PVA) Poval™ 28–99 (Kuraray Co., Ltd., Tokyo, Japan) with a degree of hydrolysis of 99–99.8 mol% and average molecular weight (MW) of 145,000 g/mol and polyacrylic acid (PAA; MW = 1800 g/mol) from Sigma-Aldrich (Saint Louis, MO, USA) were used as raw materials for the preparation of the hydrogel samples. Polyethylene glycol (PEG; MW = 400 g/mol) was also purchased from Sigma-Aldrich (Saint Louis, MO, USA).

For the drug release experiments, diclofenac sodium salt (≥98%) was obtained from Sigma-Aldrich (Saint Louis, MO, USA).

As for the hen’s egg chorioallantoic membrane test, sodium chloride (NaCl ≥ 99%) was bought from PanReac AppliChem (Darmstadt, Germany), and sodium hydroxide (NaOH ≥ 99%) was acquired from Merck (Darmstadt, Germany). Fertilized chicken eggs (≈60 g/egg) were provided by Sociedade Agrícola da Quinta da Freiria S.A. (Bombarral, Leiria, Portugal).

Working solutions were prepared in distilled and deionized (DD) water (resistivity ≥ 18 MΩ∙cm) obtained using a Milli-Q^®^ Integral 3 water purification system (Millipore, Darmstadt, Germany). Phosphate buffered saline (PBS) tablets (pH = 7.4 at 25 °C) intended for the preparation of buffered solutions were supplied by Sigma-Aldrich (Saint Louis, MO, USA).

Articular cartilage samples from the femoral condyles were obtained from a single knee joint of an adult porcine from a local slaughterhouse (Sicasal, Vila Franca do Rosário, Mafra, Portugal).

### 5.2. Methods

#### 5.2.1. Samples Preparation

##### Hydrogels

PVA hydrogels were prepared by dissolving the polymer powder in DD water (13.5% *w*/*w*) at 95 °C for 20 h, with periodic stirring to ensure the formation of a clear and homogeneous solution. A similar procedure was followed to obtain PVA/PAA gels, but PAA was added to the solution in a ratio of 3:10 (*w*/*w*) relative to PVA. In both cases, the solutions were carefully poured into glass Petri dishes (preheated at 95 °C) and cooled to room temperature for 8 h. The polymeric solutions were submitted to 6 cycles of an FT process. Freezing occurred at −20 °C for 16 h and thawing at room temperature for 8 h.

**Figure 9:**
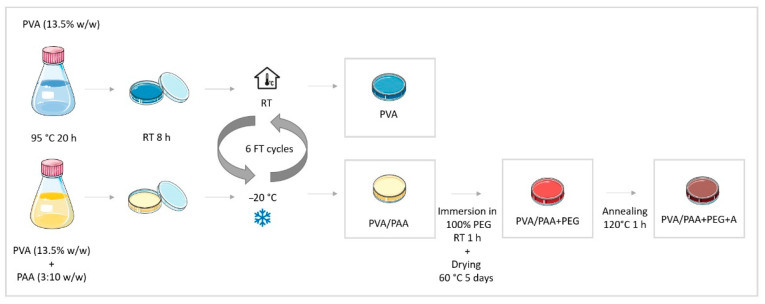
Schematic diagram of the materials’ preparation. RT: room temperature; FT: freeze-thawing.

Some PVA/PAA hydrogels were wholly immersed at room temperature in 100% PEG for 1 h. Then, they were removed from the liquid medium, blotted with absorbent paper, and dried at 60 °C for about 5 days until the weight became constant to obtain PVA/PAA+PEG hydrogels. Some of these were further subjected to an annealing (A) treatment at 120 °C for 1 h, giving rise to PVA/PAA+PEG+A hydrogels. The methodology followed for the materials’ preparation is summarized in the schematic diagram shown in Figure 9.

After production, the hydrogels were washed in DD water for 48 h (exchanged at least three times per day) and then stored hydrated till being used. All of them were opaque white and presented an average thickness of 4.8 ± 1.2 mm in the swollen state. Before each characterization test, samples were cut with the proper dimensions, rinsed with DD water, carefully cleaned with absorbent paper, and soaked in fresh DD water for at least 24 h.

##### Cartilage

The collection of the porcine knee joint was accomplished within 24 h post mortem to ensure the freshness of the tissues. Following joint dissection, full-thickness osteochondral plugs were harvested from the medial/lateral femoral condyles using a brazed hole cutter with an internal diameter of 6 mm. The underlying bone and vascularized tissue were removed from the deep zone of the cartilage plugs using a sharp scalpel, leaving the articular surface of the femoral condyles intact. Then, samples were washed with DD water and kept hydrated for 2 h at room temperature before being tested.

#### 5.2.2. Samples Characterization

##### Morphology

The surface morphology of the materials was accessed using scanning electron microscopy (SEM). The images were acquired at 15 kV using an S-2400 microscope (Hitachi Ltd., Tokyo, Japan). Before being observed, the hydrated discs (diameter 10 mm) were frozen via immersion in liquid nitrogen, broken, and then lyophilized for 24 h in an LBFD-A21 freeze dryer (Labtron, Fleet, Hampshire, UK). Subsequently, samples were coated with a conductive layer of gold/palladium (Au:Pd = 1:4) using a Q150T ES sputter coater (Quorum Technologies, Lewes, UK). At least three images of the surface microstructure and cross-cut section of each material were taken with a magnification of 3000×.

##### Water Uptake

Cylindrical samples (diameter 8 mm), fully hydrated in DD water (1 mL per sample), were dried in an oven at 37 °C for seven days. An OHAUS semi-micro analytical balance (Discovery DV215CD, Ohaus Corporation, Parsippany, NJ, USA) was used to weigh the discs in their hydrated and dry states. The swelling capacity (*SC*) was estimated through the following equation [70,71]:

(4) $$SC\left(\%\right)=\frac{\;W_{H}-W_{D}}{\;W_{D}}\times 100$$

where $W_{H}$ is the weight of hydrated samples in equilibrium and $W_{D}$ is the weight of dry samples, while the equilibrium water content (*EWC*) was determined using

(5) $$EWC\left(\%\right)=\frac{\;W_{H}-W_{D}}{\;W_{H}}\times 100$$

Each type of hydrogel was tested in triplicate. Measurements were also carried out in porcine cartilage samples (diameter 6 mm) following the same procedure.

##### Wettability

Surface wettability was assessed using the captive bubble method. The hydrated hydrogels were cut into rectangular strips (≈20 mm × 10 mm), fixed to a metal support, and immersed downward inside a quartz cell filled with DD water. Air bubbles (4.5 ± 1.5 μL) were formed beneath the material’s surface with a micrometric syringe with a J-shaped needle. Each bubble was monitored for 10 min, where it was recorded via several images at predefined times using a JAI CV-A50 video camera (JAI A/S, Grosswallstadt, Germany) mounted on an M3Z microscope (Wild, Heerbrugg, Switzerland) and connected to a DT3155 frame grabber (Data Translation, Norton, MA, USA). Image analysis was carried out with Axisymmetric Drop Shape Analysis Profile (ADSA-P) software. Three independent strips were used for each type of sample, where a total of 8–11 bubbles were analyzed. Measurements were also carried out in porcine cartilage samples following the same procedure.

##### Mechanical Behavior

The mechanical properties were evaluated through compressive tests performed in a TA.XT Express Texture Analyzer (Stable Micro Systems, Godalming, Surrey, UK) with a load cell of 50 N. Exponent Lite Express software (version 6) was used to acquire the stress–strain curves and process the results. The tests were performed with hydrated cylindrical samples (diameter 6 mm) in unconfined mode at room temperature. The discs were compressed at 0.1 mm/s to the maximum capacity of the load cell. Both loading and unloading responses were recorded. For comparison purposes, specimens (diameter 6 mm and thickness 3–4 mm) of the porcine cartilage were analyzed under the same testing conditions. Each type of sample was tested at least in quintuplicate.

##### Rheological Behavior

Rheological measurements were performed to evaluate the viscoelastic properties of the hydrated hydrogels. An MCR 92 rheometer in oscillatory mode with a parallel plate system (25 mm in diameter) was used. The gap was optimized for a 2% deformation of the hydrogels. Amplitude sweeps (γ = 0.01–100%) were carried out to determine the linear viscoelastic region (LVER) at a constant angular frequency of 10 rad/s. A strain value of γ = 0.1%, found to be within the LVER, was set during the dynamic frequency sweep tests (range: 0.1–100 rad/s) to determine the storage modulus (G’) and the loss modulus (G’’). All tests were done in quadruplicate for each hydrogel at 37 °C.

##### Friction Coefficient

The friction coefficient (CoF) of hydrogels against stainless steel was determined in reciprocal oscillating mode using a ball-on-disc geometry setup in a TRM 1000 tribometer (Wazau, Berlin, Germany). AISI 316L stainless steel spheres (Ø = 6 mm, surface roughness ≤0.1 µm), purchased from Luis Aparicio SL (Barcelona, Spain), were used in the experiments. Before testing, the hydrogel discs (diameter 65 mm) were pre-equilibrated for 24 h in the lubricating media (PBS solution). The experiments were carried out in PBS at room temperature over a stroke length of 10 mm with a tangential sliding velocity of 42 mm/s and a sliding distance of 15 m. At least three replicates were performed for each hydrogel and testing condition.

##### Drug Loading and Release

For the drug loading/release experiments, hydrogel discs (diameter 8 mm) were loaded via direct soaking of the dry samples (3 days, 37 °C) in 3 mL of a diclofenac (DFN) solution at 0.2% w/v, prepared in PBS. Once loaded, the discs were removed from the DFN solution, washed with DD water, superficially cleaned with absorbent paper, and placed in 3 mL of a PBS solution to perform the drug release assays. The experiments were done in sink conditions at 37 °C and 180 rpm using an Incubating Mini Shaker (VWR International, Alfragide, Portugal). At pre-established times, aliquots of 300 μL were taken and replaced with the same volume of fresh PBS solution. The absorbance of the collected solutions was measured at 276 nm with a Multiskan™ GO Microplate spectrophotometer (Thermo Fisher Scientific, Kandel, Germany) to estimate the amount of drug released. DFN solutions with different concentrations were prepared and their absorbance was measured at the referred wavelength to build a calibration curve. The release curves were obtained in triplicate. The methanol extraction method was applied to determine the total amount of DFN loaded into the samples [72]. The drug-loaded samples were immersed in 4 mL of pure methanol, and at pre-defined times, the supernatant methanol solution was collected and fully replaced by fresh methanol. The concentration of DFN was obtained through measurement of the absorbance of the collected solutions, as was done for the drug release measurement. The procedure was repeated till no more drug was detected in the supernatant solution.

##### HET–CAM Test

The hen’s egg test (HET) on the chorioallantoic membrane (CAM) was accomplished with fertile white leghorn chicken eggs according to the ICCVAM recommendations [73] to predict the eventual irritancy induced by PVA/PAA+PEG+A samples loaded with DFN. Although this test was originally developed to evaluate the irritability caused by materials in the eye, it can be extended to other vascularized tissues [30]. Cartilage is avascular, but the underlying bone tissue presents microvascularization, legitimizing the use of this test.

The eggs were placed in a PT-56S intelligent incubator (Jiangxi Spring Eco-Technology Co., Nanchang, Jiangxi, China) for 9 days at 37 °C and 60% relative humidity, and were automatically rotated every two hours. On the 9th day of incubation, the eggs were laid with the widest end facing upwards and a circular cut (diameter ≈ 18 mm) was made in the area of the air chamber to take out the eggshell using a Dremel^®^ 3000 rotary tool (Dremel Europe, Breda, Netherlands). Then, the inner membrane was moistened with approximately 2 mL of NaCl aqueous solution (0.9% *w*/*v*). After 30 min of incubation, the saline solution was withdrawn and the inner membrane was carefully extracted with a surgical tweezer to expose the CAM.

Hydrated hydrogel samples (diameter 8 mm) were directly placed on the eggs’ CAM and left for 5 min. Negative and positive controls were prepared, pouring 300 μL of aqueous solutions of NaCl (0.9% *w*/*v*) or NaOH (0.1 M), respectively, into the eggs’ CAM. The membranes were monitored for the appearance of any signs of hemorrhage, vascular lysis, or coagulation, and the times it took for such injuries to occur (*tH*, *tL*, and *tC* measured in seconds) were recorded. The irritation score (*IS*) was calculated as follows [74]:

(6) $$IS=\frac{\left(301-tH\right)}{300}\times 5+\frac{\left(301-tL\right)}{300}\times 7+\frac{\left(301-tC\right)}{300}\times 9$$

Samples are classified as non-irritating if *IS* < 1, mildly irritating if 1 ≤ *IS* < 5, moderately irritating if 5 ≤ *IS* < 10, or severely irritating if *IS* > 10. For each experimental condition, three eggs were used.

##### Statistics

IBM^®^ SPSS^®^ Statistics software (version 25 from IBM Corporation, Armonk, NY, USA) was used to perform the statistical analysis. Quantitative measurements are expressed in terms of mean ± standard deviation. Normality was verified using Shapiro–Wilk test and the homogeneity of variance using Levene’s test. When both were verified, one-way ANOVA was applied, followed by Tukey’s HSD post hoc test for multiple comparisons. Data that showed a normal distribution with unequal variances were analyzed using Welch’s ANOVA and Dunnett’s T3 post hoc test to determine significant differences between groups. An independent samples *t*-test was also used to compare means between two groups of data with a normal distribution when appropriate. A *p*-value lower than 0.05 was considered significant. All statistical differences between the data sets are indicated by asterisks (* *p* < 0.05, ** *p* < 0.005, *** *p* < 0.0005). Unless stated otherwise, the asterisks are placed graphically on top of each group, referring to the comparison with the pair of the corresponding color.

## Figures and Tables

**Table 1:** DFN loading capacity into the samples and percentage of drug released (average values ± standard deviations, *n* = 4).

	PVA	PVA/PAA	PVA/PAA+PEG	PVA/PAA+PEG+A
DFN amount loaded (μg DFN/mg dry sample)	14.6 ± 0.9	125.5 ± 9.2	77.9 ± 4.8	81.7 ± 11.6
DFN released (%)	57.8 ± 3.6	45.7 ± 3.3	64.3 ± 4.0	55.3 ± 7.9

## Data Availability

The raw data required for these findings are available upon request via email to andreia.oliveira@tecnico.ulisboa.pt.

## References

[1] Katz J. (2019). Promoting Clinical and Basic Research in Osteoarthritis. OARSI World Congr. Osteoarthr. Promot. Clin. Basic Res. Osteoarthr..

[2] Yelin E., Weinstein S., King T. (2016). The Burden of Musculoskeletal Diseases in the United States. Seminars in Arthritis and Rheumatism.

[3] Vahedi P., Moghaddamshahabi R., Webster T.J., Koyuncu A.C.C., Ahmadian E., Khan W.S., Mohamed A.J., Eftekhari A. (2021). The use of infrapatellar fat pad-derived Mesenchymal stem cells in Articular cartilage regeneration: A review. Int. J. Mol. Sci..

[4] Shirley P.Y., Hunter D.J. (2015). Managing osteoarthritis. Aust. Prescr..

[5] Chuang E.Y., Chiang C.W., Wong P.C., Chen C.H. (2018). Hydrogels for the Application of Articular Cartilage Tissue Engineering: A Review of Hydrogels. Adv. Mater. Sci. Eng..

[6] Freedman B.R., Mooney D.J. (2019). Biomaterials to Mimic and Heal Connective Tissues. Adv. Mater..

[7] Sefat F., Israr Raja T., Sohai lZafar M., Khurshid Z., Najeeb S., Zohail S., Ahmadi E.D., Rahmati M., Mozafari M. (2019). Chapter 3—Nanoengineered biomaterials for cartilage repair. Nanoengineered Biomaterials for Regenerative Medicine.

[8] Bicho D., Pina S., Reis R.L., Oliveira J.M. (2018). Commercial Products for Osteochondral Tissue Repair and Regeneration. Adv. Exp. Med. Biol..

[9] Rongen J.J., van Tienen T.G., van Bochove B., Grijpma D.W., Buma P. (2014). Biomaterials in search of a meniscus substitute. Biomaterials.

[10] Spiller K.L., Maher S.A., Lowman A.M. (2011). Hydrogels for the repair of articular cartilage defects. Tissue Eng.—Part B Rev..

[11] Li J., Mooney D.J. (2016). Designing hydrogels for controlled drug delivery. Nat. Rev. Mater..

[12] Oliveira A.S., Seidi O., Ribeiro N., Colaço R., Serro A.P. (2019). Tribomechanical comparison between PVA hydrogels obtained using different processing conditions and human cartilage. Materials.

[13] Eftekhari A., Dizaj S.M., Sharifi S., Salatin S., Saadat Y.R., Vahed S.Z., Samiei M., Ardalan M., Rameshrad M., Ahmadian E. (2020). The use of nanomaterials in tissue engineering for cartilage regeneration; current approaches and future perspectives. Int. J. Mol. Sci..

[14] Chamkouri1 H., Chamkouri M. (2021). A Review of Hydrogels, Their Properties and Applications in Medicine. Am. J. Biomed. Sci. Res..

[15] Peppas N.A., Hassan C.M. (2000). Structure and applications of poly (vinyl alcohol) hydrogels produced by conventional crosslinking or by freezing/thawing methods. Biopolymers—PVA Hydrogels, Anionic Polymerisation Nanocomposites.

[16] Moreau D. (2016). Design and Characterization of Hydrogel Films and Hydrogel-Ceramic Composites for Biomedical Applications. Doctoral Dissertation.

[17] Kawanishi K., Komatsu M., Inoue T. (1987). Thermodynamic consideration of the sol-gel transition in polymer solutions. Polymer.

[18] Kanca Y., Milner P., Dini D., Amis A.A. (2018). Tribological properties of PVA/PVP blend hydrogels against articular cartilage. J. Mech. Behav. Biomed. Mater..

[19] Wu X., Li W., Chen K., Zhang D., Xu L., Yang X. (2019). A tough PVA/HA/COL composite hydrogel with simple process and excellent mechanical properties. Mater. Today Commun..

[20] Luo X., Akram M.Y., Yuan Y., Nie J., Zhu X. (2019). Silicon dioxide/poly(vinyl alcohol) composite hydrogels with high mechanical properties and low swellability. J. Appl. Polym. Sci..

[21] Barbon S., Contran M., Stocco E., Todros S., Macchi V., De Caro R., Porzionato A. (2021). Enhanced biomechanical properties of polyvinyl alcohol-based hybrid scaffolds for cartilage tissue engineering. Processes.

[22] Jiang Y., Yang Y., Zheng X., Yi Y., Chen X., Li Y., Sun D., Zhang L. (2020). Multifunctional load-bearing hybrid hydrogel with combined drug release and photothermal conversion functions. NPG Asia Mater..

[23] Gonzalez J.S., Alvarez V.A. (2011). The effect of the annealing on the poly(vinyl alcohol) obtained by freezing-thawing. Thermochim. Acta.

[24] Rey-Rico A., Madry H., Cucchiarini M. (2016). Hydrogel-Based Controlled Delivery Systems for Articular Cartilage Repair. Biomed Res. Int..

[25] Westin C.B., Nagahara M.H.T., Decarli M.C., Kelly D.J., Moraes Â.M. (2020). Development and characterization of carbohydrate-based thermosensitive hydrogels for cartilage tissue engineering. Eur. Polym. J..

[26] Gao Y., Li K., Guo L., Fan H., Fan Y., Zhang X. (2020). Fabrication of biomimetic hydrogel for chondrocyte delivery. Mater. Lett..

[27] Joshi N., Yan J., Levy S., Bhagchandani S., Slaughter K.V., Sherman N.E., Amirault J., Wang Y., Riegel L., He X. (2018). Towards an arthritis flare-responsive drug delivery system. Nat. Commun..

[28] Jung Y.S., Park W., Park H., Lee D.K., Na K. (2017). Thermo-sensitive injectable hydrogel based on the physical mixing of hyaluronic acid and Pluronic F-127 for sustained NSAID delivery. Carbohydr. Polym..

[29] Petit A., Sandker M., Müller B., Meyboom R., van Midwoud P., Bruin P., Redout E.M., Versluijs-Helder M., van der Lest C.H.A., Buwalda S.J. (2014). Release behavior and intra-articular biocompatibility of celecoxib-loaded acetyl-capped PCLA-PEG-PCLA thermogels. Biomaterials.

[30] Oliveira A.S., Schweizer S., Nolasco P., Barahona I., Saraiva J., Colaço R., Serro A.P. (2020). Tough and low friction polyvinyl alcohol hydrogels loaded with anti-inflammatories for cartilage replacement. Lubricants.

[31] Lindgren U., Djupsjö H. (1985). Diclofenac for pain after hip surgery. Acta Orthop..

[32] Cosmo G., Congedo E. (2015). The Use of NSAIDs in the Postoperative Period: Advantage and Disadvantages. J. Anesth. Crit. Care Open Access.

[33] Gunaydin C., Bilge S.S. (2018). Effects of nonsteroidal anti-inflammatory drugs at the molecular level. Eurasian J. Med..

[34] Filipe H.P., Henriques J., Reis P., Silva P.C., Quadrado M.J., Serro A.P. (2016). Contact lenses as drug controlled release systems: A narrative review. Rev. Bras. Oftalmol..

[35] Franco P., De Marco I. (2021). Contact lenses as ophthalmic drug delivery systems: A review. Polymers.

[36] Jeong J.O., Park J.S., Kim E.J., Jeong S.I., Lee J.Y., Lim Y.M. (2020). Preparation of radiation cross-linked poly(Acrylic acid) hydrogel containing metronidazole with enhanced antibacterial activity. Int. J. Mol. Sci..

[37] Vasi A.M., Popa M.I., Tanase E.C., Butnaru M., Verestiuc L. (2014). Poly(Acrylic Acid)-Poly(Ethylene Glycol) nanoparticles designed for ophthalmic drug delivery. J. Pharm. Sci..

[38] Kobayashi M., Koide T., Hyon S.H. (2014). Tribological characteristics of polyethylene glycol (PEG) as a lubricant for wear resistance of ultra-high-molecular-weight polyethylene (UHMWPE) in artificial knee join. J. Mech. Behav. Biomed. Mater..

[39] Zhang S., Hu F., Li J., Lv L., Lu H. (2021). Lubrication Effect of Polyvinyl Alcohol/Polyethylene Glycol Gel for Artificial Joints. Adv. Mater. Sci. Eng..

[40] Davis J., McLister A. (2016). Passive and Interactive Dressing Materials. Smart Bandage Technologies.

[41] Choi J., Kung H.J., MacIas C.E., Muratoglu O.K. (2012). Highly lubricious poly(vinyl alcohol)-poly(acrylic acid) hydrogels. J. Biomed. Mater. Res.—Part B Appl. Biomater..

[42] Ricciardi R., Auriemma F., De Rosa C. (2005). Structure and properties of poly(vinyl alcohol) hydrogels obtained by freeze/thaw techniques. Macromol. Symp..

[43] Teodorescu M., Morariu S., Bercea M. (2016). Advanced Materials Based on Multicomponent Polymeric Systems. Multiphase Polymer Systems.

[44] Sanchez L.M., Alvarez V.A. (2019). Development of potentially biocompatible hydrogels with cylindrical pores prepared from polyvinyl alcohol and low-molecular weight polyacrylic acid. Polym. Eng. Sci..

[45] Muratoglu O.K., Choi J., Bodugoz-Senturk H., Braithwaite G.J.C., Spiegelberg S.H. (2016). Tough Hydrogels. Patent.

[46] Choi J., Muratoglu O.K., Choi J., Muratoglu O.K. (2008). PVA-PAA Hydrogels. Patent.

[47] Liu P., Chen W., Liu C., Tian M., Liu P. (2019). A novel poly (vinyl alcohol)/poly (ethylene glycol) scaffold for tissue engineering with a unique bimodal open-celled structure fabricated using supercritical fluid foaming. Sci. Rep..

[48] Park J.S., Kim H.A., Choi J.B., Gwon H.J., Shin Y.M., Lim Y.M., Khil M.S., Nho Y.C. (2012). Effects of annealing and the addition of PEG on the PVA based hydrogel by gamma ray. Radiat. Phys. Chem..

[49] Sophia Fox A.J., Bedi A., Rodeo S.A. (2009). The basic science of articular cartilage: Structure, composition, and function. Sports Health.

[50] Arakawa C.K., DeForest C.A. (2017). Polymer Design and Development. Biology and Engineering of Stem Cell Niches.

[51] Bhalani D.V., Trivedi J.S., Jewrajka S.K. (2021). Selective grafting of morphologically modified poly(vinylidene fluoride) ultrafiltration membrane by poly(acrylic acid) for inducing antifouling property. Appl. Surf. Sci..

[52] Wu J., Zhao C., Lin W., Hu R., Wang Q., Chen H., Li L., Chen S., Zheng J. (2014). Binding characteristics between polyethylene glycol (PEG) and proteins in aqueous solution. J. Mater. Chem. B.

[53] Pawlak Z., Petelska A.D., Urbaniak W., Yusuf K.Q., Oloyede A. (2013). Relationship Between Wettability and Lubrication Characteristics of the Surfaces of Contacting Phospholipid-Based Membranes. Cell Biochem. Biophys..

[54] Ahmed A.S., Mandal U.K., Taher M., Susanti D., Jaffri J.M. (2018). PVA-PEG physically cross-linked hydrogel film as a wound dressing: Experimental design and optimization. Pharm. Dev. Technol..

[55] Shi Y., Xiong D., Li J., Li L., Liu Q., Dini D. (2021). Tribological Rehydration and Its Role on Frictional Behavior of PVA/GO Hydrogels for Cartilage Replacement under Migrating and Stationary Contact Conditions. Tribol. Lett..

[56] Liu T., Jiao C., Peng X., Chen Y.N., Chen Y., He C., Liu R., Wang H. (2018). Super-strong and tough poly(vinyl alcohol)/poly(acrylic acid) hydrogels reinforced by hydrogen bonding. J. Mater. Chem. B.

[57] Tanaka E., Rego E.B., Iwabuchi Y., Inubushi T., Koolstra J.H., Van Eijden T.M.G.J., Kawai N., Kudo Y., Takata T., Tanne K. (2008). Biomechanical response of condylar cartilage-on-bone to dynamic shear. J. Biomed. Mater. Res.—Part A.

[58] Perni S., Prokopovich P. (2020). Rheometer enabled study of cartilage frequency-dependent properties. Sci. Rep..

[59] Li F., Su Y., Wang J., Wu G., Wang C. (2010). Influence of dynamic load on friction behavior of human articular cartilage, stainless steel and polyvinyl alcohol hydrogel as artificial cartilage. J. Mater. Sci. Mater. Med..

[60] Oungoulian S.R., Durney K.M., Jones B.K., Ahmad C.S., Hung C.T., Ateshian G.A. (2015). Wear and damage of articular cartilage with friction against orthopedic implant materials. J. Biomech..

[61] Siboro S.A.P., Anugrah D.S.B., Ramesh K., Park S.H., Kim H.R., Lim K.T. (2021). Tunable porosity of covalently crosslinked alginate-based hydrogels and its significance in drug release behavior. Carbohydr. Polym..

[62] Sanli O., Asman G. (2003). Release of Diclofenac through Gluteraldehyde Crosslinked Poly (vinyl alcohol)/Poly (acrylic acid). J. Appl. Polym. Sci..

[63] Jalil A., Khan S., Naeem F., Haider M.S., Sarwar S., Riaz A., Ranjha N.M. (2017). The structural, morphological and thermal properties of grafted ph-sensitive interpenetrating highly porous polymeric composites of sodium alginate/acrylic acid copolymers for controlled delivery of diclofenac potassium. Des. Monomers Polym..

[64] Chen R., Li G., Han A., Wu H., Guo S. (2016). Controlled release of diclofenac sodium from polylactide acid-based solid dispersions prepared by hot-melt extrusion. J. Biomater. Sci. Polym. Ed..

[65] Ritger P.L., Peppas N.A. (1987). A simple equation for description of solute release I. Fickian and non-fickian release from non-swellable devices in the form of slabs, spheres, cylinders or discs. J. Control. Release.

[66] Rezaei A., Nasirpour A. (2019). Evaluation of Release Kinetics and Mechanisms of Curcumin and Curcumin-β-Cyclodextrin Inclusion Complex Incorporated in Electrospun Almond Gum/PVA Nanofibers in Simulated Saliva and Simulated Gastrointestinal Conditions. Bionanoscience.

[67] Flory P.J., Rehner J. (1943). Statistical mechanics of cross-linked polymer networks II. Swelling. J. Chem. Phys..

[68] Kuijpers A.J., Engbers G.H.M., Feijen J., De Smedt S.C., Meyvis T.K.L., Demeester J., Krijgsveld J., Zaat S.A.J., Dankert J. (1999). Characterization of the network structure of carbodiimide cross-linked gelatin gels. Macromolecules.

[69] Pimenta A.F.R., Ascenso J., Fernandes J.C.S., Colaço R., Serro A.P., Saramago B. (2016). Controlled drug release from hydrogels for contact lenses: Drug partitioning and diffusion. Int. J. Pharm..

[70] Pal K., Banthia A.K., Majumdar D.K. (2009). Polymeric hydrogels: Characterization and biomedical applications. Des. Monomers Polym..

[71] Gibas I., Janik H. (2010). Review: Synthetic Polymer Hydrogels for Biomedical Applications. Chem. Chem. Technol..

[72] Silva D., de Sousa H.C., Gil M.H., Santos L.F., Oom M.S., Alvarez-Lorenzo C., Saramago B., Serro A.P. (2021). Moxifloxacin-imprinted silicone-based hydrogels as contact lens materials for extended drug release. Eur. J. Pharm. Sci..

[73] ICCVAM (2010). The Hen’s Egg Test—Chorioallantoic Membrane Test Method. ICCVAM Test Method Evaluation Report: Current Validation Status of In Vitro Test Methods Proposed for Identifying Eye Injury Hazard Potential of Chemicals and Products.

[74] Loftsson T., Stefánsson E. (2017). Cyclodextrins and topical drug delivery to the anterior and posterior segments of the eye. Int. J. Pharm..

